# Editorial Comment to Immune Checkpoint Inhibitor Retreatment after Enfortumab Vedotin in Advanced Urothelial Carcinoma: A Descriptive Study with Exploratory Evaluation of Radiotherapy

**DOI:** 10.31662/jmaj.2026-0081

**Published:** 2026-07-10

**Authors:** Jimpei Miyakawa

**Affiliations:** 1 Department of Urology, Graduate School of Medicine, The University of Tokyo, Tokyo, Japan

**Keywords:** immune checkpoint inhibitor, advanced urothelial carcinoma, radiotherapy, combination therapy, enfortumab vedotin

Systemic therapy for advanced urothelial carcinoma involves three types of treatment and their combinations: platinum-based chemotherapy (PBC), immune checkpoint inhibitors (ICI), and enfortumab vedotin (EV). EV monotherapy is approved as a third-line treatment following PBC and ICI therapy—evidence for treatment after EV is scarce. Though fibroblast growth factor receptor 3 (FGFR3) inhibitors represent an option, their applicability is limited to specific cases. The National Comprehensive Cancer Network (NCCN) guidelines recommend “biomarker-guided therapy” as the preferred regimen, citing the FGFR3 inhibitor erdafitinib and trastuzumab deruxtecan for human epidermal growth factor 2 (HER2)-positive cases. For patients not eligible for these options, gemcitabine or taxane monotherapy may be considered; however, the evidence level for these is low, and they are not established treatments ^(1)^.

Minato et al. ^(2)^ presented a paper on third-line EV treatment for advanced urothelial carcinoma. They summarize the therapeutic efficacy of re-administering ICI after EV, combined with concurrent radiation therapy to the main progressive lesion or symptomatic sites. It is noteworthy that they achieved disease control with ICI rechallenge in cases where initial ICI therapy provided a long-term response and in cases with lymph node metastasis only. For cases where initial ICI therapy failed, PBS may be a preferable alternative. However, these were essentially end-stage cases; though local control with radiation therapy was good, its contribution to overall survival was unclear ^(2)^.

In the retrospective study by Maiorano et al. ^(3)^, better outcomes were observed in cases where the initial ICI rechallenge was for nonmetastatic urothelial carcinoma (UC) and where the interval between the end of the initial ICI and rechallenge was 12 months or longer. The longest duration of response was achieved with the anti-Programmed death-1 (PD-1) antibody following the anti-Programmed death ligand 1 (PD-L1) antibody ^(3)^.

Makrakis et al. ^(4)^ reported 25 cases of advanced UC rechallenged with ICI; the objective response rate to the second ICI was only 16%, and the disease control rate was 48%. Although interpretation is difficult due to the small number of cases and variability in background factors and treatment regimen, second-line ICI can be expected to have some efficacy in certain patient populations ^(4)^.

Radiation therapy is known not only to shrink tumors at the irradiation site but also to induce the abscopal effect, where distant metastases not exposed to radiation shrink. Combining radiation therapy with ICI amplifies the abscopal effect ^(5)^. Cases have been reported where efficacy was restored after radiation therapy, even in patients where ICI had become ineffective ^(6)^. Although radiation therapy for metastatic UC generally does not prolong survival and is considered palliative treatment, it holds potential for achieving certain therapeutic effects when combined with ICI.

Since the publication of the EV-302 trial, the treatment strategy for advanced UC has undergone significant changes ^(7)^. EV plus pembrolizumab is used as first-line therapy, followed by PBC. However, the treatment approach after completion of EV, ICI, and PBC remains unestablished. Rechallenge with ICI combined with radiotherapy may become a viable treatment option under certain conditions. A larger accumulation of cases is necessary to consider treatment options beyond third-line therapy.

## Article Information

### Conflicts of Interest

None

## References

[1] NCCN clinical practice guidelines in oncology: bladder cancer. version 3. 2025;2025.

[2] Minato A, Sugita Y, Mizushima Y, et al. Immune checkpoint inhibitor retreatment after enfortumab vedotin in advanced urothelial carcinoma: a descriptive study with exploratory evaluation of radiotherapy. JMA J. 2026;9(4):1001-1011. 10.31662/jmaj.2025-0463 PMC13453616 42577015

[3] Maiorano BA, Cigliola A, Tateo V, et al. Outcomes of immune checkpoint inhibitor rechallenge in advanced urothelial carcinoma: results from a global real-world evidence study. ESMO Open. 2025;10(12):105862. 41259899 10.1016/j.esmoop.2025.105862 PMC12670551

[4] Makrakis D, Bakaloudi DR, Talukder R, et al. Treatment rechallenge with immune checkpoint inhibitors in advanced urothelial carcinoma. Clin Genitourin Cancer. 2023;21(2):286-94. 36481176 10.1016/j.clgc.2022.11.003

[5] Rompré-Brodeur A, Shinde-Jadhav S, Ayoub M, et al. PD-1/PD-L1 immune checkpoint inhibition with radiation in bladder cancer: in situ and abscopal effects. Mol Cancer Ther. 2020;19(1):211-20. 31534011 10.1158/1535-7163.MCT-18-0986

[6] Postow MA, Callahan MK, Barker CA, et al. Immunologic correlates of the abscopal effect in a patient with melanoma. N Engl J Med. 2012;366(10):925-31. 22397654 10.1056/NEJMoa1112824 PMC3345206

[7] Powles T, Valderrama BP, Gupta S, et al. Enfortumab vedotin and pembrolizumab in untreated advanced urothelial cancer. N Engl J Med. 2024;390(10):875-88. 38446675 10.1056/NEJMoa2312117

